# Hypergammaglobulinemia before Starting DAA Therapy Is A Strong Predictor of Disease Progression in Cirrhotic Patients Even after HCV Clearance

**DOI:** 10.3390/jpm12111794

**Published:** 2022-10-31

**Authors:** Maria Stella Franzè, Roberto Filomia, Gaia Caccamo, Concetta Pitrone, Angela Alibrandi, Carlo Saitta, Amalia Rita Caspanello, Clelia Asero, Vittoria Arcadi, Giovanni Raimondo, Irene Cacciola

**Affiliations:** 1 Department of Clinical and Experimental Medicine, University of Messina, 98124 Messina, Italy; 2 Division of Medicine and Hepatology, Department of Internal Medicine, University Hospital of Messina, 98124 Messina, Italy; 3 Department of Economics Unit of Statistical and Mathematical Science of Messina, University of Messina, 98124 Messina, Italy

**Keywords:** hypergammaglobulinemia, DAA treatment, liver cirrhosis, HCV clearance, hepatocellular carcinoma, liver decompensation

## Abstract

The predictive factors of long-term clinical benefits in patients with hepatitis C virus (HCV)—related liver cirrhosis after Direct Antiviral Agents (DAA) treatment are still undefined. The aim of this study was to identify any predictors of liver failure, hepatocellular carcinoma (HCC) and/or death in patients with compensated liver cirrhosis who achieved the sustained virological response (SVR). To this purpose, 324 consecutive cirrhotic patients who started DAA treatment from 1 April 2015 to 31 December 2016 were retrospectively analyzed. All patients were followed up for a median time of 63 months (range 19–77) through clinical/biochemical/instrumental examinations performed at baseline and after stopping the DAA treatment. At the end of the evaluation, 230 (71%) individuals showed stable clinical liver disease over time, 43 (13.3%) developed HCC, and 24 (7.4%) developed hepatic decompensation without HCC. Overall, 49 (15,1%) patients died. Multivariate regression analysis showed that hepatic decompensation was significantly associated with at baseline older age, higher liver stiffness, higher spleen longitudinal size values and hypergammaglobulinemia (*p* = 0.003, *p* = 0.005, *p* = 0.001, *p* = 0.029, respectively). HCC development was significantly associated with hypergammaglobulinemia (*p* < 0.001). Death was associated with older age and hypergammaglobulinemia (*p* < 0.001 and *p* = 0.007, respectively). Finally, survival analysis confirmed that patients with gamma globulin levels ≥ 1.8 gr/dl had a significantly higher risk of death compared to those with gamma globulin levels < 1.8 gr/dl (*p* < 0.001). In conclusion, hypergammaglobulinemia before starting DAA therapy represents a strong predictor of hepatic decompensation, HCC and death in cirrhotic patients even after HCV clearance.

## 1. Introduction

In the last decade, the availability of direct-acting antiviral drugs (DAAs) to treat hepatitis C virus (HCV) infection has radically changed the natural history of liver disease in HCV-positive patients [1,2,3].

In fact, the effectiveness, the easy handling and the few side effects of DAA treatment have allowed the treatment of HCV-positive patients even with advanced liver disease [4,5], reducing the risk of liver-related events, including hepatocellular carcinoma (HCC), hepatic decompensation, as well as liver-related mortality [6,7,8,9,10,11,12,13,14,15].

Despite this evidence, it is important to underline that there exists a non-negligible percentage of patients in whom liver disease inexorably progresses after the eradication of HCV infection, even in the absence of other comorbidities, including hepatitis B virus (HBV) co-infection, alcohol abuse or decompensated diabetes. In fact, the risk of HCC occurrence could also persist due to the underlying chronic liver disease. However, to date, the real prevalence of patients “cured” from HCV infection that showed a progression of the liver disease after a long follow-up and the identification of any predictive factors of liver decompensation or of the stability of the disease are still missing.

In this context, it seems of considerable clinical importance to identify the possible predictive factors of liver disease progression.

The aim of this study was to evaluate the clinical/biochemical/instrumental parameters at the time of starting DAA therapy and during the subsequent long-lasting follow-up after the end of DAA treatment, with the final objective of finding any possible predictor of liver failure, HCC development and/or death in patients with liver cirrhosis cured from HCV infection.

## 2. Patients and Methods

### 2.1. Patients

The study aimed to retrospectively evaluate the prevalence of liver decompensation, HCC and death in all consecutive compensated cirrhotic patients (Child-Pugh A) who started DAA treatment from 1 April 2015 to 31 December 2016 at the Medicine and Hepatology Unit of the University Hospital of Messina.

Exclusion criteria were: previous liver transplant, diagnosis of HCC in the six months before the start of DAA treatment, HBV or immunodeficiency virus co-infection and signs of decompensated liver cirrhosis (i.e., ascites, portosystemic encephalopathy and hyperbilirubinemia) at the time of start DAA therapy. At baseline, diagnosis and staging of the liver disease were performed according to the established clinical, ultrasonographic, elastographic, endoscopic and biochemical criteria [5], and confirmed by histology in the cases that had undergone liver biopsy in that time period. The interferon-free, DAA-based anti-HCV therapies have been available in Italy since April 2015, and they were prescribed in accordance with the indications of the Italian Agency of the Drug (AIFA).”

According to the “AIFA prescription limitations, all cirrhotic patients had liver stiffness values higher than 12 kPa at FibroScan evaluation. Additional criteria for making a diagnosis of cirrhosis were a previous liver biopsy showing stage 4 fibrosis, presence of esophageal and/or gastric varices at endoscopy examination, platelet count lower than 100 × 10^3^/mmc, and typical ultrasound features [16,17,18,19]. The baseline was defined at the time of starting DAA therapy. The DAAs are identified as protease or polymerase inhibitors, and they interfere at different steps of HCV replication.

Sustained viral eradication (SVR) was defined as an undetectable HCV-RNA level, as assessed by highly sensitive molecular methods, 12 weeks after the end of DAA treatment. Antiviral treatment was administered at the physician’s discretion, according to drug labels and international recommendations [20,21]. Each patient underwent clinical, biochemical, ultrasonographic and esophago-gastro-duodenoscopic examinations performed at baseline and after stopping the DAA treatment according to international guidelines. In particular, all patients underwent regular follow-up through clinical and biochemical examinations performed every 3–6 months, an abdominal ultrasound performed every 6 months and esophago-gastro-duodenoscopy (EGDS) scheduled at intervals according to international guidelines [22,23,24,25,26,27,28].

According to the design of the study, the following data were collected from each patient and recorded in a dataset at baseline: demographic data [age, sex, body mass index (BMI)], presence of comorbidities [type 2 diabetes mellitus (T2DM), arterial hypertension, cryoglobulinemic vasculitis, depressive syndrome, chronic renal failure, ischemic cardiopathy, hemoglobinopathy, lymphoproliferative/myeloproliferative syndrome, monoclonal gammopathy], virological data (type of DAA therapy and HCV genotype), liver stiffness values, presence and size of esophageal varices, echo-graphic parameters (portal vein diameter, longitudinal spleen size), hepatitis B core antibody (anti-HBc) positivity, hematological and biochemical data [hemoglobin (Hb) values, counts of white blood cells (WBC) and platelets (PLT), values of serum aspartate aminotransferase (AST), alanine aminotransferase (ALT), bilirubin, albumin and gamma globulins, creatinine and international normalized ratio (INR)]. All hematological and biochemical data were collected at the basal time and at the last date of follow-up available. Drinking habits in all patients were also investigated and quantified as the number of units drunk [one drink or alcoholic unit (AU) = 12.5 gr of pure ethanol contained in a glass of wine, a pint of beer or a small glass of spirits]. The subjects were thus identified as non-consumers, drinkers of less than 2 AU, and drinkers of more than 2 AU [29]. Moreover, the withdrawal of drinking habits was also registered.

For the evaluation of the clinical outcomes, we stratified the patients considering: (1) onset of hepatocellular carcinoma; (2) onset of progression of the liver disease, including progression or bleeding of esophageal varices, encephalopathy and ascites in the absence of hepatocellular carcinoma; (3) death. Patients in whom none of these events occurred were considered stable patients over time.

Finally, we also stratified the patients on the basis of resisting HCV scores [RESIST-HCV low risk (LR) if they had PLT count >120,000/L and serum albumin 3.6 g/dL or RESIST-HCV high risk (HR) if they had PLT count <120,000/L or serum albumin <3.6 g/dL] to Monitor Progression of Esophageal Varices [30,31,32].

The study was performed in accordance with the principles of the Declaration of Helsinki, and it was approved by the ethical committee of the district of Messina.

### 2.2. Statistical Analysis

Continuous numerical variables are expressed as medians and interquartile ranges, whereas categorical variables as numbers and rates. Examined continuous variables did not present normal distribution as verified using the Kolmogorov–Smirnov test, and the non-parametric approach was consequently used. The Mann–Whitney U test was used to compare patients with and without stable liver disease during follow-up for continuous variables. The Chi-square or Fisher’s exact tests (where appropriate) were applied for categorical variables. Univariate Logistic Regression Models were estimated to assess the possible dependence of the outcomes of interest (development of HCC, decompensating event, death/OLT) by all the examined variables at baseline (sex, age, BMI, type 2 diabetes mellitus, arterial hypertension, liver stiffness, portal vein diameter, longitudinal spleen size, anti-HBc positivity, AST, ALT, bilirubin, INR, PLT, albumin, gamma globulin and creatinine). Multivariate logistic regression models were estimated, including variables resulting in significant univariate analyses. Survival curves were plotted using the Kaplan–Meier method, and comparisons between subgroups were performed using the long-rank test. Survival curves of patients with compensated cirrhosis at presentation included patients who developed HCC during the study period. The Cox regression model was used to identify prognostic factors of survival. Statistical analysis was performed using SPSS software version 26.0 for Windows package (SPSS Inc., IBM Company, Chicago, IL, USA); 2-sided *p* < 0.05 was considered to be statistically significant.

## 3. Results

### 3.1. Patients’ Characteristics

**Figure 1:**
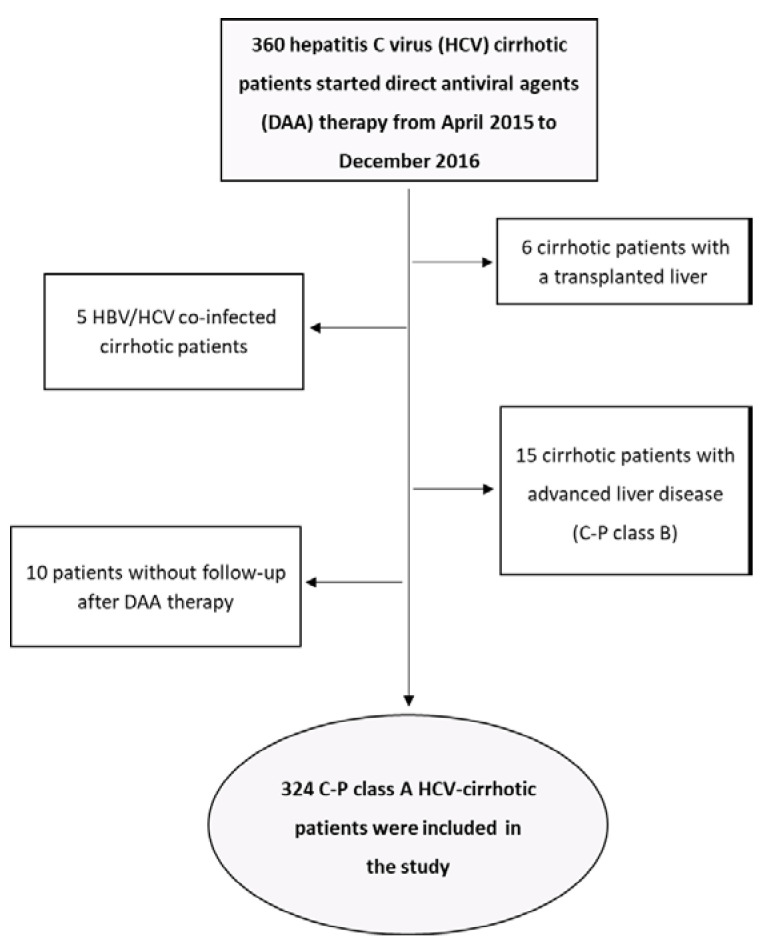
Flow chart of 324 C-P class HCV cirrhotic patients included in the study.

Three hundred and 65 Child-Pugh (C-P) class A cirrhotic patients consecutively observed in our Liver Unit have started HCV DAA treatment from 1 April 2015 to 31 December 2016. Five of them (1.4%) were excluded because of HBV coinfection, six (1.6%) because they had undergone orthotopic liver transplantation (OLT), 15 (4.1%) because they had signs of liver decompensation at the start of DAA treatment (Child-Pugh class B), and 10 (2.7%) because they were lost to follow-up after the end of DAA treatment. Therefore, a total number of 324 patients [172 male/152 female; median age 67 years (range 57–75)] were included in the study (Figure 1). All patients were followed up for a median time of 63 months (range 19–77) by clinical, biochemical and instrumental examinations. Baseline demographic, clinical and laboratory characteristics of the included patients are reported in Table 1.

One-hundred and thirty-two patients (40.7%) started DAA therapy with Sofosbuvir/Ledipasvir, 75 (23.1%) Sofosbuvir plus Daclatasvir, 61 (18.8%) Sofosbuvir plus Simeprevir, 28 (8.6%) Sofosbuvir and 28 (8.6%) Ombitasvir/Paritaprevir/Ritonavir plus Dasabuvir. Ribavirin was added in 118 (36.4%) cases. The distribution of HCV-genotype was the following: 30 cases (9.3%) with HCV-genotype 1a, 200 (61.7%) genotype 1b, 47 (14.5%) genotype 2, 20 (6.2%) genotype 3, and 27 (8.3%) genotype 4. Moreover, ninety-nine patients (30.6%) had a previous HBV infection with anti-HBc positivity. Two hundred and five patients (63.3%) had non-drinking habits, 73 (22.5%) were drinkers of less than 2 AU, and 46 (14.2%) were drinkers of more than 2 AU. Thirty-one patients (63.3%) stopped drinking habits before basal time. One-hundred and eighty patients (55.6%) had arterial hypertension, 96 (29.6%) had type 2 diabetes mellitus, 39 (12%) were affected by depressive syndromes, 16 (4.9%) had chronic renal failure, 11 (3.4%) presented cryoglobulinemic vasculitis, eight (2.5%) had ischemic cardiopathy, and 22 (6.7%) had hematological disorders (11 hemoglobinopathies, seven lymphoproliferative syndromes, and four monoclonal gammopathies).

The esophagogastroduodenoscopy was available for 260 patients (80.2%) before starting DAA therapy. At baseline, 99 up to 260 patients (38.1%) showed gastric-esophageal varices: 88 patients (33.8%) had small size varices (F1), nine (3.5%) had medium size varices (F2), and two (0.8%) had large size varices (F3). Fourteen patients (4.3%) had a previous diagnosis of HCC, with complete response after treatment and without recurrence in the six months before the time of starting DAA therapy. Thirty patients (9.3%) had a history of hepatic decompensation, which resolved before enrollment in the study. In particular, 15 patients (4.6%) had a previous ascites decompensation, nine (2.8%) had a previous episode of hepatic encephalopathy, and eight (2.5%) had previous gastroesophageal varices bleeding.

During the follow-up, 43 out of 324 (13.3%) patients developed HCC, while 24 (7.4%) developed hepatic decompensation without HCC. Overall, 49/324 patients (15.1%) died at a median time of 36 months (range 19–53 months): among them, 23 (7.1%) had HCC, 9 (2.8%) had liver decompensation without HCC, and 17 (5.2%) had an extrahepatic disease.

In addition, we also evaluated the grade of esophageal varices during the follow-up. Among the 99 patients who had esophageal varices at baseline, 7 (7.1%) showed an increased size of esophageal varices, 58 (58.6%) maintained stably-sized esophageal varices, and 21 patients (21.2%) reduced the size of esophageal varices. Finally, 10 (3.1%) patients without evidence of esophageal varices at baseline developed them during follow-up without any other signs of progression of liver disease. According to a recent study, we also evaluate the reduction of portal hypertension by applying the “resist HCV criteria”: we stratified the patients on the basis of RESIT HCV score, and our results confirm that Resist-HCV HR score was associated with a progression of esophageal varices^32^.

Overall, 230 out of 324 patients (71%) showed a stable disease over time. According to the study design, we firstly conducted the statistical analysis comparing the group of patients with stable diseases over time to the patients who have had a progression of liver disease. The analyses showed that there were no statistically significant differences concerning the median time of follow-up between patients with stable liver disease over time and patients who developed hepatic decompensation [median time of follow-up 63 months (41–77) vs. 62 months (38–73), *p* = 0.27].

No statistically significant differences concerning gender distribution, type 2 diabetes mellitus, arterial hypertension, anti-HBc positivity, BMI, AST values, ALT values and bilirubin were observed at the basal time between the two subsets of patients. On the contrary, patients who developed liver events during follow-up had higher age (*p* = 0.01), presence and higher sizes of esophageal varices (*p* < 0.0001), higher liver stiffness values (*p* < 0.0001), higher portal vein and spleen longitudinal diameters (*p* = 0.004, *p* < 0.0001, respectively), higher rate of mortality (*p* < 0.0001), higher INR values (*p* < 0.0001), higher creatinine values (*p* = 0.01), higher gamma globulin amount (*p* < 0.0001), lower albumin and lower counts of platelets (*p* = 0.014 and *p* < 0.0001, respectively), than patients who had a stable disease over time (Table 2).

### 3.2. Analysis of the Predictors of Liver Decompensation Development, HCC and Death

In order to assess the possible association between the variables examined and the progression of liver disease, we performed univariate and multivariate logistic regression analyses of different variables with respect to (1) progression of liver disease HCC-free, (2) occurrence of HCC and (3) death.

In detail, we examined the possible association, as independent predictors of liver decompensation, of all the variables evaluated at baseline of the DAA treatment: the univariate logistic regression analysis showed that the development of hepatic decompensation was associated with older age (*p* = 0.003), higher values of liver stiffness (*p* = 0.005), portal vein diameter (*p* = 0.001), spleen longitudinal size (*p* < 0.0001), INR (*p* = 0.035), higher values of serum gamma globulins (*p* < 0.0001) and lower values of ALT and PLT (*p* = 0.025, *p* = 0.003, respectively). Older age (*p* = 0.003, O.R. 1.072, 95% C.I. 1.025–1.122), higher liver stiffness values (*p* = 0.005, O.R. 1.045, 95% C.I. 1.013–1.073), higher spleen longitudinal size (*p* = 0.001, O.R. 1.383, 95% C.I. 1.143–1.674) and higher values of serum gamma-globulins (*p* = 0.029, O.R. 2.511, 95% C.I. 1.101–5.726) maintained the significant association with development of liver decompensation at the multivariate regression analysis (Table 3).

The univariate logistic regression analysis showed that HCC development was associated with higher liver stiffness (*p* = 0.017), higher gamma globulin amounts (*p* < 0.0001), lower BMI (*p* = 0.026) and PLT count (*p* = 0.034). The multivariate regression analysis confirmed that higher levels of serum gamma-globulin were independent predictors of the development of HCC (*p* < 0.0001, O.R. 4.182, 95% C.I. 2.014–8.685) during follow-up (Table 4).

At univariate logistic regression analysis, death was associated with older age (*p* = 0.001), higher liver stiffness (*p* = 0.02), higher portal vein diameter (*p* = 0.014), higher INR values (*p* = 0.047) and higher levels of gamma-globulins (*p* < 0.0001) before starting DAA therapy. Only older age (*p* < 0.0001, O.R. 1.076, 95% C.I. 1.034–1.119) and increased gamma globulin values (*p* = 0.007, O.R. 2.653, 95% C.I. 1.300–5.415) were confirmed as independent predictors of death at multivariate analysis (Table 5).

Cox-regression analysis showed that male gender (*p* = 0.008, Hazard Ratio 4.198, 95% C.I. 1.459–12.083) and gamma globulin values ≥ 1.8 gr/dl (*p* = 0.039, Hazard Ratio 2.929, 95% C.I. 1.044–5.030) at baseline were significant independent predictors of death.

**Figure 2:**
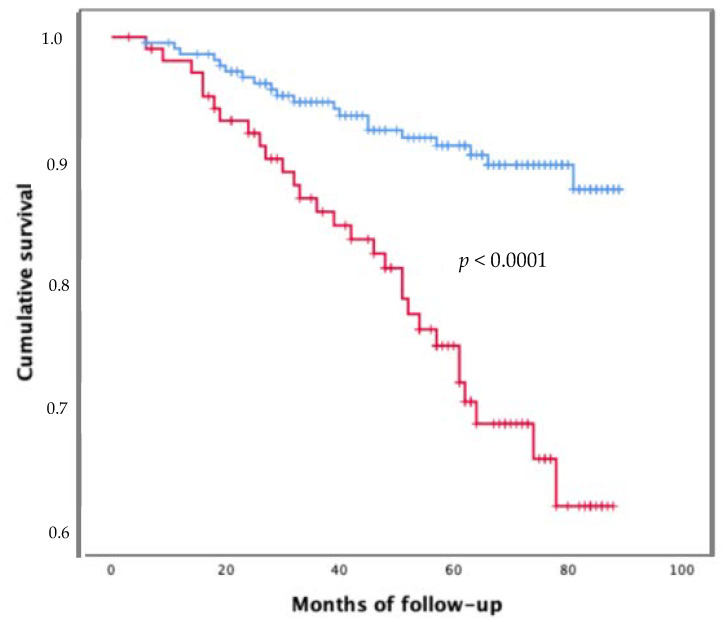
Survival curves of 324 C-P class A cirrhotic patients with gamma globulin < 1.8 gr/dl (blue line) and ≥ 1.8 gr/dl (red line).

**Figure 3:**
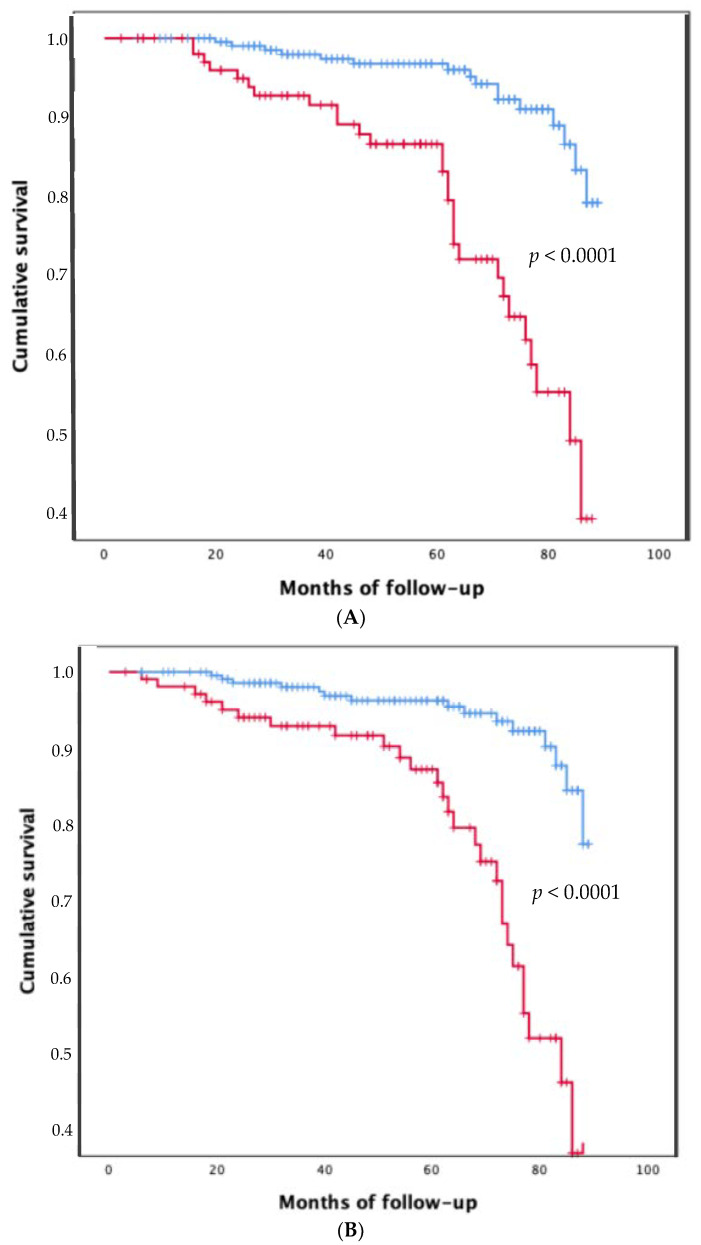
Incidence of liver decompensation events (**A**) and HCC development (**B**) in patients with gamma globulin <1.8 gr/dl (blue line) and ≥ 1.8 gr/dl (red line).

The Kaplan–Meier survival analysis confirmed that patients with gamma globulin levels ≥ 1.8 gr/dl had a significantly higher risk of death compared to those with gamma globulin levels < 1.8 gr/dl (*p* < 0.0001; Figure 2). Of note, patients with hypergammaglobulinemia at baseline decreased the gamma globulin values over time and after the end of DAA treatment. Indeed, the gamma globulin values were significantly higher at baseline than at the end of the follow-up (*p* < 0.0001). Furthermore, comparing the other biochemical parameters, there was a statistically significant difference between the baseline and the end of the follow-up for GOT (*p* < 0.0001) and GPT (*p* < 0.0001) values, PLT (*p* < 0.0001), INR values (*p* = 0.008), serum albumin (*p* < 0.0001) and creatinine (*p* < 0.0001) values. Finally, the time of occurrence of both liver decompensation events and HCC development was significantly shorter in patients with gamma globulin values ≥ 1.8 g/dL (*p* < 0.0001 and *p* < 0.0001, respectively) than patients with gamma globulin values < 1.8 g/dL (Figure 3A,B).

## 4. Discussion

The exceptional efficacy and tolerability of direct-acting antiviral drugs for the treatment of HCV infection have changed the global scenario of virus-related liver disease. However, it is important to emphasize that, particularly in patients with liver cirrhosis, recovery from the infection does not always correspond to recovery from liver disease [33,34,35,36]. And therefore, achieving SVR cannot be considered a safe surrogate marker for the clinical efficacy of DAA treatment in all stages of liver disease.

Some important questions still remain to be clarified: above all, establish the real prevalence of liver disease progression (as decompensation or HCC) in these patients; second, identify the possible factors related to disease progression.

This study showed a different spectrum of clinical events in patients with compensated cirrhosis cured from HCV infection and followed up for a long time.

Relevantly, the results of the study showed that about a quarter of the patients (77 individuals), followed up for a median time of 63 months, had a progression of the liver disease despite the HCV eradication and 31 of them died. In particular, 43 (13.6%) developed HCC, 24 patients (7.4%) had liver decompensation, and 10 had a progression of esophageal varices without other signs of progression of the liver disease.

Some important information has risen from the analysis of quite a large number of routinely available parameters collected at the time of cirrhosis diagnosis.

Comparing the population of patients with progression of liver disease with those with the stable disease over time, our results showed that there is no statistically significant difference between the two groups of patients regarding the time of follow-up, sex distribution, BMI, the prevalence of diabetes and hypertension, AT and Bilirubin values.

On the contrary, patients who developed liver complications were those with older age and biochemical or instrumental signs of more serious liver disease, including higher gamma globulin values, lower platelets values, lower albumin values, higher liver stiffness values, as well as higher spleen longitudinal size and Resist-HCV score HR.

The multivariate logistic regression analysis showed that the progression of liver disease was associated with gamma globulin ≥ 1.8 gr/dl, higher liver stiffness values and higher spleen longitudinal size; male gender and gamma globulin ≥ 1.8 gr/dl were associated with death, and only gamma globulin ≥ 1.8 gr/dl is associated with a higher risk of developing HCC.

Of note, gamma globulin levels ≥ 1.8 gr/dl represent the only variable significantly associated with both a 4.5-times greater risk of developing HCC, a 2.5-times greater risk of developing hepatic decompensation and with a 2.9-times greater risk of death. These results are in line with (in according to) a previous study that demonstrated that hypergammaglobulinemia was predictive of Child-Pugh score progression over time in patients with HCV or cryptogenic cirrhosis and that patients with increased gamma-globulin levels had a higher risk of developing HCC and death [37].

Moreover, hypergammaglobulinemia at baseline remained a strong predictor of death at Kaplan–Meier survival analysis in cirrhotic patients “cured” from HCV infection.

The main strength of our study is linked to the possibility of following a large cohort of patients from eastern Sicily and Calabria for a long time. According to previous studies, in the 1st year after the achievement of the sustained virological response (SVR), the prevalence of hepatic events is very low (about 4%), and then it gradually increases up to 20% after 5 years of follow-up. This result reinforces the importance of continuing the follow-up of cirrhotic patients over time because the risk of disease progression, understood as both hepatic decompensation and death, increases over time [3].

## 5. Conclusions

The study indicates that the detection of gamma-globulins values >1.8 gr/dl before starting DAA therapy may identify patients with a high risk of liver disease progression and/or hepatocellular carcinoma even after HCV clearance. Therefore, the gamma-globulin test could be a useful cheap prognostic tool to be routinely used in patients “cured” of HCV infection.

## Figures and Tables

**Table 1:** Baseline demographic, clinical and laboratory characteristics of 324 C-P class A HCV cirrhotic patients included in the study.

Age, Years (Range)	67 (57–75)
Male, *n* (%)	172 (53.1)
Median follow-up, months	63 (40.3–77)
BMI, Kg/m^2^	25.9 (24–28.6)
Liver stiffness, Kpa	21 (15.6–30.7)
Portal vein diameter, mm	12 (11–12.5)
Longitudinal size of the spleen, cm	13 (11.4–14.5)
Type 2 diabetes mellitus, *n* (%)	96 (29.6)
Arterial hypertension, *n* (%)	180 (55.6)
Gastro-esophageal varices *	
Absent, *n* (%)	161 (49.7)
Small size, *n* (%)	88 (27.2)
Medium size, *n* (%)	9 (2.8)
Large size, *n* (%)	2 (0.6)
Anti-HBc positivity, *n* (%)	99 (30.6)
AST, U/L	69 (44–97)
ALT, U/L	74 (45.3–118.5)
Total bilirubin, mg/dL	0.8 (0.6–1.1)
INR	1.1 (1–1.2)
PLT, mmc × 10^3^	136 (100.3–177)
Albumin, gr/dL	3.8 (3.6–4.1)
Gamma-globulins, gr/dL	1.5 (1.3–1.9)
Creatinine, mg/dL	0.9 (0.7–1)
Death, *n* (%)	49 (15.1)

All numerical parameters are expressed as median and interquartile range. BMI, body mass index; ALT, alanine aminotransferase; AST, aspartate aminotransferase; INR, international normalized ratio; PLT, platelets. * Esophago-gastro-duodenoscopy (EGDS) was available in 260/324 patients at baseline.

**Table 2:** Baseline demographic, clinical and laboratory characteristics of 307 C-P class A HCV cirrhotic patients with stable liver disease (230 cases) and patients that developed liver events (77 cases).

	Patients with Stable Liver Disease (*n* = 230)	Patients with Liver Events (*n* = 77)	*p*
Age, years (range)	66 (55–73)	69 (61–77)	0.01
Male, *n* (%)	125 (53)	39 (54.9)	0.77
Median follow-up, months	65 (42– 79)	62 (40–74)	0.27
BMI, Kg/m^2^	26 (24.2–28.8)	26.1 (23.7–28.3)	0.84
Liver stiffness, Kpa	20 (14.6–26.3)	29 (17.5–44.3)	<0.0001
Portal vein diameter, mm	12 (11–12.5)	12 (12–13)	0.004
Longitudinal size of the spleen, cm	12.7 (11.3–14)	14 (12.3–16)	<0.0001
Type 2 diabetes mellitus, *n* (%)	68 (28.8)	20 (28.2)	0.92
Arterial hypertension, *n* (%)	129 (54.7)	40 (56.3)	0.80
Gastro-esophageal varices *			<0.0001
Absent, *n* (%)	122 (68.9)	28 (41.2)	
Small size, *n* (%)	51 (28.8)	33 (48.5)	
Medium size, *n* (%)	4 (2.3)	5 (7.4)	
Large size, *n* (%)	0 (0)	2 (2.9)	
Anti-HBc positivity, *n* (%)	77 (32.6)	19 (26.8)	0.35
AST, U/L	698(44–96)	73 (50–125)	0.11
ALT, U/L	74 (48–123.7)	75 (48–121)	0.98
Total bilirubin, mg/dL	0.8 (0.6–1)	0.8 (0.6–1.2)	0.06
INR	1.0 (1–1.1)	1.1 (1.0–1.2)	<0.0001
PLT, mmc × 10^3^	142 (114–185)	101 (68–147)	<0.0001
Albumin, gr/dL	3.9 (3.7–4.1)	3.7 (3.5–4.1)	0.01
Gamma-globulins, gr/dL	1.4 (1.2–1.7)	0.9 (0.6–1.2)	<0.0001
Creatinine, mg/dL	0.8 (0.6–1)	1.9 (1.5–2.2)	0.01
Death, *n* (%)	6 (2.5)	26 (36.6)	<0.0001

All numerical parameters are expressed as median and interquartile range. Bold characters identify statistically significant results. BMI, body mass index; ALT, alanine aminotransferase; AST, aspartate aminotransferase; INR, international normalized ratio; PLT, platelets. * EGDS was available in 245/307 patients at baseline.

**Table 3:** Univariate and multivariate logistic regression analyses of different predictors variables of hepatic decompensation.

	Univariate Model			Multivariate Model		
Variables	O.R.	95% C.I.	*p*	O.R.	95% C.I.	*p*
Sex (female vs. male)	1.351	0.699–2.610	0.371	-	-	-
Age	1.039	1.004–1.074	0.027	1.072	1.025–1.122	0.003
BMI, Kg/m^2^	0.989	0.926–1.056	0.746	-	-	-
Type 2 diabetes mellitus	0.943	0.461–1.930	0.872	-	-	-
Arterial hypertension	1.077	0.560–2.073	0.824	-	-	-
Liver stiffness, Kpa	1.053	1.029–1.078	<0.0001	1.042	1.013–1.073	0.005
Portal vein diameter (mm)	1.501	1.192–1.891	0.001	1.172	0.887–1.549	0.265
Spleen longitudinal size (cm)	1.361	1.186–1.562	<0.0001	1.383	1.143–1.674	0.001
Anti-HBc positivity	0.782	0.376–1.627	0.511	-	-	-
AST, U/L	0.997	0.990–1.005	0.455	-	-	-
ALT, U/L	0.992	0.984–0.999	0.025	0.992	0.983–1.000	0.058
Bilirubin, mg/dl	1.508	0.855–2.661	0.156	-	-	-
INR	7.811	1.150–53.062	0.035	1.568	0.242–10.138	0.637
PLT, mmc × 10^3^	0.990	0.984–0.997	0.003	2.511	0.995–1.011	0.424
Albumin, gr/dl	0.612	0.256–1.458	0.267	-	-	-
Gamma-globulins (<1.8 vs. ≥1.8), gr/dl	4.625	2.337–9.153	<0.0001	2.511	1.101–5.726	0.029
Creatinine, mg/dl	2.761	0.830–9.177	0.098	-	-	-

Bold characters identify statistically significant results in the multivariate analysis. O.R., odds ratio; C.I., confidence interval; BMI, body mass index; PLT, platelets; AST, aspartate aminotransferase; ALT, alanine aminotransferase; INR, international normalized ratio.

**Table 4:** Univariate and multivariate logistic regression analyses of different predictors variables of hepatocellular carcinoma (HCC).

	Univariate Model			Multivariate Model		
Variables	O.R.	95% C.I.	*p*	O.R.	95% C.I.	*p*
Sex (female vs. male)	1.583	0.817–3.066	0.173	-	-	-
Age	1.023	0.991–1.055	0.164	-	-	-
BMI, Kg/m^2^	0.903	0.826–0.988	0.026	0.911	0.824–1.008	0.070
Type 2 diabetes mellitus	0.908	0.445–1.853	0.791	-	-	-
Arterial hypertension	1.412	0.729–2.735	0.307	-	-	-
Liver stiffness, Kpa	1.028	1.005–1.051	0.017	1.009	0.984–1.035	0.470
Portal vein diameter (mm)	1.122	0.912–1.380	0.277	-	-	-
Spleen longitudinal size (cm)	1.094	0.959–1.248	0.183	-	-	-
Anti-HBc positivity	0.863	0.423–1.761	0.686	-	-	-
AST, U/L	1.004	0.998–1.010	0.188	-	-	-
ALT, U/L	1.000	0.994–1.006	0.996	-	-	-
Bilirubin, mg/dl	1.508	0.855–2.661	0.156	-	-	-
INR	1.644	0.942–2.867	0.080	-	-	-
PLT, mmc × 10^3^	0.994	0.988–1.000	0.034	0.335	0.992–1.003	0.335
Albumin, gr/dl	0.580	0.245–1.373	0.215	-	-	-
Gamma globulin (<1.8 vs. ≥1.8), gr/dl	4.858	2.463–9.583	<0.0001	4.182	2.014–8.685	<0.0001
Creatinine, mg/dl	3.106	0.948–10.171	0.061	-	-	-

Bold characters identify statistically significant results in the multivariate analysis. O.R., odds ratio; C.I., confidence interval; BMI, body mass index; PLT, platelets; AST, aspartate aminotransferase; ALT, alanine aminotransferase; INR, international normalized ratio.

**Table 5:** Univariate and multivariate logistic regression analyses of different predictors variables of death.

	Univariate Model			Multivariate Model		
Variables	O.R.	95% C.I.	*p*	O.R.	95% C.I.	*p*
Sex (female vs. male)	1.059	1.024–1.086	0.001	1.076	1.034–1.119	<0.0001
Age	1.479	0.794–2.753	0.217	-	-	-
BMI, Kg/m^2^	0.945	0.874–1.021	0.152	-	-	-
Type 2 diabetes mellitus	1.629	0.866–3.064	0.130	-	-	-
Arterial hypertension	1.615	0.857–3.046	0.138	-	-	-
Liver stiffness, Kpa	1.027	1.004–1.050	0.020	1.022	0.995–1.050	0.116
Portal vein diameter (mm)	1.285	1.052–1.569	0.014	0.425	0.869–1.395	0.425
Spleen longitudinal size (cm)	1.118	0.987–1.267	0.079	-	-	-
Anti-HBc positivity	0.794	0.401–1.572	0.507	-	-	-
AST, U/L	1.002	0.996–1.008	0.608	-	-	-
ALT, U/L	0.996	0.990–1.002	0.181	-	-	-
Bilirubin, mg/dl	1.472	0.853–2.540	0.164	-	-	-
INR	5.956	1.022–34.717	0.047	1.483	0.282–7.786	0.641
PLT, mmc × 10^3^	0.997	0.992–1.002	0.242	-	-	-
Albumin, g/dl	0.449	0.196–1.031	0.059	-	-	-
Gamma globulin (<1.8 vs. ≥1.8), g/dl	3.729	1.991–6.984	<0.0001	2.653	1.300–5.415	0.007
Creatinine, mg/dl	1.427	0.436–4.668	0.557	-	-	-
